# Absence of CD36 alters systemic vitamin A homeostasis

**DOI:** 10.1038/s41598-020-77411-5

**Published:** 2020-11-23

**Authors:** Michael J. Trites, Maria Febbraio, Robin D. Clugston

**Affiliations:** 1 Department of Physiology, University of Alberta, 7-49 Medical Sciences Building, Edmonton, AB T6G 2H7 Canada; 2 Group on the Molecular and Cell Biology of Lipids, Faculty of Medicine and Dentistry, University of Alberta, Edmonton, AB Canada; 3 Department of Dentistry, Faculty of Medicine and Dentistry, University of Alberta, Edmonton, AB Canada

**Keywords:** Metabolism, Lipids

## Abstract

Fatty acid translocase (CD36) is a scavenger receptor with multiple ligands and diverse physiological actions. We recently reported that alcohol-induced hepatic retinoid mobilization is impaired in *Cd36*^*−/−*^ mice, leading us to hypothesize that CD36 has a novel role in hepatic vitamin A mobilization. Given the central role of the liver in systemic vitamin A homeostasis we also postulated that absence of CD36 would affect whole-body vitamin A homeostasis. We tested this hypothesis in aging wild type and *Cd36*^*−/−*^ mice, as well as mice fed a vitamin A-deficient diet. In agreement with our hypothesis, *Cd36*^*−/−*^ mice accumulated hepatic retinyl ester stores with age to a greater extent than wild type mice. However, contrary to expectations, *Cd36*^*−/−*^ mice consuming a vitamin A-deficient diet mobilized hepatic retinoid similar to wild type mice. Interestingly, we observed that *Cd36*^*−/−*^ mice had significantly reduced white adipose tissue retinoid levels compared to wild type mice. In conclusion, we demonstrate that the absence of CD36 alters whole-body vitamin A homeostasis.

## Introduction

Vitamin A is an essential micronutrient that is acquired from the diet either as preformed vitamin A or as vitamin A precursors, such as β-carotene^1^. Vitamin A specifically denotes all-*trans*-retinol however the term vitamin A is typically used to encompass additional retinoid metabolites including retinoic acid, retinaldehyde, and retinyl ester^2^. All-*trans*-retinoic acid is the primary active vitamin A metabolite acting as a ligand for nuclear receptors influencing expression of over 500 genes involved in diverse biological processes including cell differentiation, proliferation, apoptosis, vision, reproduction, and immunity^3^. Due to its potency retinoic acid concentrations in the body are tightly controlled and represent less than 1% of the total retinoid content in the body^4^. Eleven-*cis*-retinal is a major component of chromophores in the retina and is critical for its role in vision and thus has higher-concentrations in the eye, however elsewhere in the body retinaldehyde species primarily serve as metabolic intermediates in the conversion of retinol to retinoic acid^5^. The most abundant forms of vitamin A in the body are retinol and retinyl esters. Retinyl esters are the primary storage form of vitamin A, accounting for 80–85% of the body’s total vitamin A content, being localized to specialized lipid droplets in hepatic stellate cells (HSCs)^4,6,7^. When required by extrahepatic tissues vitamin A is mobilized from HSC’s by being transferred to hepatocytes and then released from the liver bound to retinol binding protein 4 (RBP4). Retinol is then transported throughout the body in the circulation providing the body with a continuous supply of vitamin A^8,9^. Although the mobilization of vitamin A from the liver to the circulation has been well studied over recent decades significant questions remain in our understanding of this intricate process^10^. Notably, the loss of vitamin A stores from the liver is a common feature in several models of liver disease including non-alcoholic fatty liver disease, cirrhosis, hepatocellular carcinoma, and alcoholic liver disease^11^. Interestingly, we recently discovered that loss of fatty acid translocase (CD36) protects mice from alcoholic steatosis and allows them to retain their vitamin A stores suggesting a direct link between CD36 and hepatic vitamin A mobilization^12–14^.

CD36 is a transmembrane glycoprotein that is a scavenger receptor capable of binding several ligands, including long-chain fatty acids, lipoproteins, and oxidized lipids^15–18^. CD36 has also been implicated in several physiological functions such as lipid homeostasis, immune defense, and angiogenesis^15^. Given its central role in fatty acid (FA) uptake it is not surprising that expression of CD36 is highly skewed towards tissues specialized for FA storage and oxidation, specifically adipose and cardiac tissue, respectively^19^. One of the major phenotypes accompanying global CD36 deletion is highlighted by increased circulating FAs due to the reduced ability of adipose, cardiac, and skeletal muscle to uptake FAs^16,20^. Subsequent investigation using tissue-specific ablation of CD36 has yielded further understanding of the diverse roles CD36 plays in maintaining physiological homeostasis^19,21–24^. CD36 expression is considered low in the liver, however CD36 is expressed by the entire population of liver cells, including hepatocytes, HSCs, Kuppfer cells, and endothelial cells and is increased in patients with non-alcoholic- and alcoholic-liver disease^25–27^.

In this report we aimed to investigate the link between CD36 and hepatic retinoid metabolism. Given the central role of the liver in regulating systemic vitamin A homeostasis we also explored the role of CD36 in whole-body vitamin A metabolism. Given that *Cd36*^*−/−*^ mice were protected against alcohol-induced hepatic retinoid loss we hypothesized that absence of CD36 would prevent mobilization of hepatic vitamin A^13^. We further postulated that impaired hepatic vitamin A mobilization would subsequently affect extrahepatic tissues.

## Results

### *Cd36*^*−/−*^ mice have altered whole-body vitamin A homeostasis

Our initial hypothesis was that the altered hepatic vitamin A phenotype in *Cd36*^*−/−*^ mice noted in our previous work would also have an effect on whole-body vitamin A homeostasis^13^. We postulated that *Cd36*^*−/−*^ mice would accumulate hepatic vitamin A and have impaired retinoid mobilization, similar to *Rbp4*^*−/−*^ mice^13^. We thus investigated tissue vitamin A levels throughout the life span of wild type (WT) and *Cd36*^*−/−*^ mice (20 days, 3-, 6-, and 9-months). The physical characteristics of WT and *Cd36*^*−/−*^ mice were consistent with previous reports (Table 1)^28^. Specifically, *Cd36*^*−/−*^ mice had reduced body weight, reduced white adipose tissue (WAT) mass, and similar absolute liver mass compared to their WT counterparts, however when normalized to body weight *Cd36*^*−/−*^ mice had relatively larger livers.

**Table 1:** Physical characteristics of WT and *Cd36*^*−/−*^ mice throughout their lifespan.

	Body weight (g)^a,b^	Liver weight (g)^b^	Liver:body weight ratio^a,b^	White adipose weight (g)^a,b^	White adipose:body ratio^a,b^
**20 days**					
Wild type (8)	10.98 ± 0.74	0.62 ± 0.05	0.056 ± 0.001	0.055 ± 0.013	0.005 ± 0.001
*Cd36*^*−/−*^ (7)	9.22 ± 1.13*	0.47 ± 0.08	0.051 ± 0.006*	0.029 ± 0.008*	0.003 ± 0.001*
**3 months**					
Wild type (9)	27.76 ± 1.84	1.39 ± 0.12	0.050 ± 0.002	0.510 ± 0.13	0.018 ± 0.004
*Cd36*^*−/−*^ (7)	29.15 ± 0.86	1.73 ± 0.10*	0.059 ± 0.002*	0.448 ± 0.14	0.015 ± 0.005*
**6 months**					
Wild type (11)	33.52 ± 2.51	1.53 ± 0.17	0.046 ± 0.005	1.15 ± 0.36	0.034 ± 0.008
*Cd36*^*−/−*^ (7)	31.25 ± 1.65	1.69 ± 0.19	0.054 ± 0.004*	0.77 ± 0.20*	0.024 ± 0.005*
**9 months**					
Wild type (9)	36.11 ± 2.16	1.91 ± 0.16	0.053 ± 0.004	1.40 ± 0.50	0.038 ± 0.012
*Cd36*^*−/−*^ (6)	33.04 ± 1.76*	1.73 ± 0.13	0.053 ± 0.006	0.82 ± 0.16*	0.025 ± 0.004

The *n* number for each experimental group is provided in parentheses in the first column. All data are presented as mean ± SD. Data analyzed by two-way ANOVA.

*Post-test significantly different between genotypes within mice of the same age.

^a^Significant genotype effect.

^b^Significant age effect.

**Figure 1:**
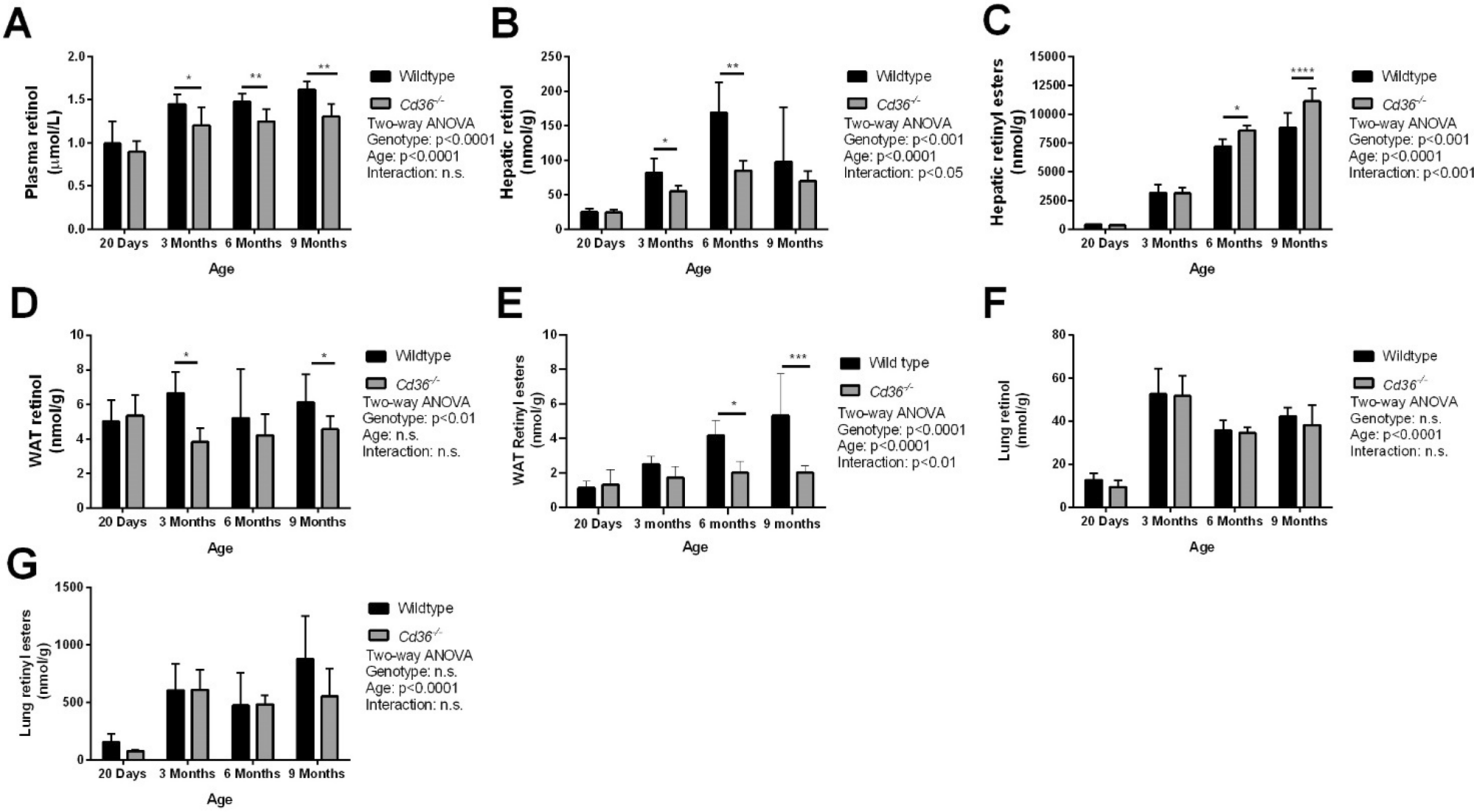
*Cd36*^*−/−*^ mice have altered whole-body vitamin A homeostasis. HPLC was used to determine tissue levels of (**A**) plasma retinol, (**B**) hepatic retinol, (**C**) hepatic retinyl esters, (**D**) WAT retinol, (**E**) WAT retinyl esters, (**F**) lung retinol, (**G**) lung retinyl esters. Tissue retinoid levels were normalized to volume of plasma (**A**) or tissue mass (**B**–**G**). Data is shown as mean ± S.D. and significance determined by two-way ANOVA (n = 6 per group). *p < 0.05, **p < 0.01, and ****p < 0.0001 denote a significant post-test result between genotypes within mice of the same age.

High performance liquid chromatography (HPLC) analysis of plasma and hepatic retinoids agreed with our previous reports^13^, where *Cd36*^*−/−*^ mice had reduced plasma retinol but increased hepatic retinoid as determined by two-way ANOVA (Fig. 1A–C). Hepatic retinol levels were significantly lower in *Cd36*^*−/−*^ mice (Fig. 1B) however, we observed a significant age-dependent accumulation of the more abundant retinyl ester storage form (Fig. 1C), primarily driven by an increase in retinyl esters in *Cd36*^*−/−*^ mice at 6- and 9-months of age compared to WT mice. Second only to the liver, WAT is a significant site of vitamin A accumulation^4^. Interestingly, WAT retinoid levels were lower in *Cd36*^*−/−*^ mice (Fig. 1D,E). Reduced WAT retinoid was driven by a two-pronged effect: *Cd36*^*−/−*^ mice had significantly reduced tissue retinol that was unchanged with age (Fig. 1D) and significantly reduced tissue retinyl esters that were exacerbated with age (Fig. 1E). Although a relatively minor contributor to whole-body vitamin A stores, the lungs are capable of storing retinyl esters in pulmonary stellate cells, similar to the liver^29^. We observed no significant difference between WT and *Cd36*^*−/−*^ mice in regard to lung retinoid levels but observed an age-dependent increase in both retinol and retinyl ester, primarily driven by a large increase between 20 days and 3-months (Fig. 1F,G). These data indicate that absence of CD36 affects whole-body vitamin A homeostasis.

**Figure 2:**
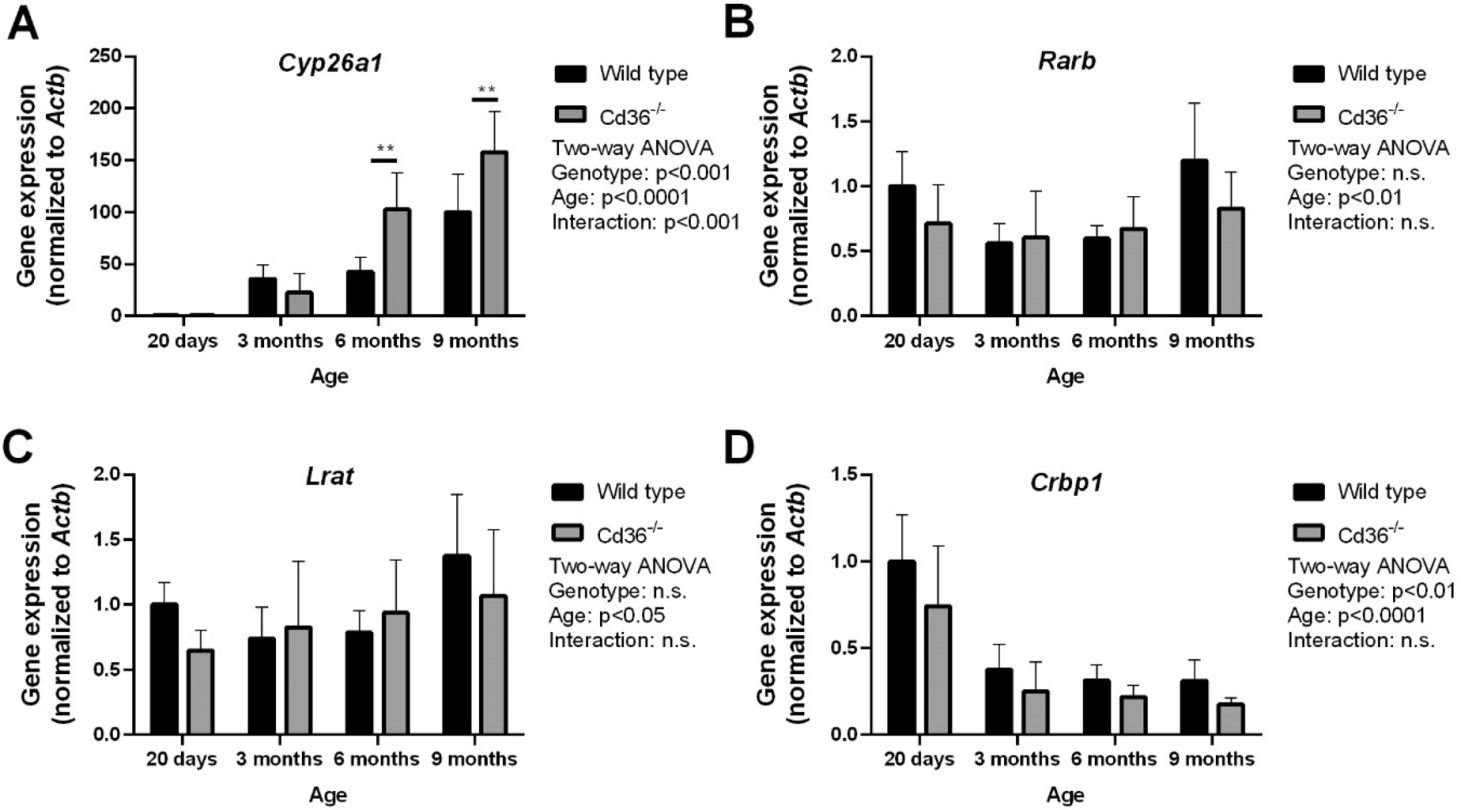
Hepatic gene expression levels of key mediators of vitamin A metabolism in aging wild type and *Cd36*^*−/−*^ mice. qPCR was used to determined relative hepatic gene expression levels of (**A**) *Cyp26a1*, (**B**) *Rarb*, (**C**) *Lrat*, (**D**) *Crbp1*. Data is shown as mean ± S.D. and significance determined by two-way ANOVA (n = 6 per group). *p < 0.05 denotes a significant post-test result between genotypes within mice of the same age.

To further examine the altered retinoid phenotype in the liver of *Cd36*^*−/−*^ mice we examined hepatic gene expression of key mediators of retinoid metabolism (Fig. 2). *Cyp26a1* is the primary retinoic acid hydroxylase in the liver being responsible for the breakdown and disposal of retinoic acid^30,31^. Following the increased hepatic retinoid content, *Cd36*^*−/−*^ mice had significantly greater expression of hepatic *Cyp26a1* at 6- and 9-months of age with both WT and *Cd36*^*−/−*^ mice displaying an age-dependent increase in *Cyp26a1* expression (Fig. 2A). Hepatic *Rarb*, which encodes the beta-isoform of the nucleic retinoic acid receptor, has also been shown to be sensitive to hepatic retinoic acid levels^32^. WT and *Cd36*^*−/−*^ mice had similar expression levels of hepatic *Rarb* and both demonstrated an age-dependent increase in hepatic *Rarb* expression (Fig. 2B). We examined hepatic *Lrat* expression as it encodes lecithin:retinol acyltransferase (LRAT), which is the sole enzyme responsible for retinyl ester synthesis in the liver^6^. We observed similar hepatic *Lrat* expression levels between WT and *Cd36*^*−/−*^ mice and an age-dependant increase in hepatic *Lrat* expression in both genotypes (Fig. 2C). Interestingly, hepatic *Crbp1* expression had a reciprocal pattern to hepatic retinoid levels. *Crbp1* encodes the gene for cellular retinoid binding protein, which is one of the primary intracellular retinoid binding proteins responsible for chaperoning retinoid throughout the cell^33^. Specifically, *Cd36*^*−/−*^ mice had significantly reduced hepatic *Crbp1* expression and both WT and *Cd36*^*−/−*^ mice displayed an age-dependant decrease in hepatic *Crbp1* expression (Fig. 2D).

### *Cd36*^*−/−*^ mice are able to defend against systemic vitamin A deficiency on a vitamin A deficient diet

To further explore our hypothesis that CD36 has a role in facilitating hepatic vitamin A mobilization we placed WT and *Cd36*^*−/−*^ on a vitamin A deficient diet to promote mobilization of hepatic vitamin A stores. We hypothesized that if CD36 is required for mobilization of hepatic vitamin A then *Cd36*^*−/−*^ mice placed on a diet devoid of vitamin A would be unable to draw on hepatic vitamin A stores to maintain circulating retinol levels and would rapidly display a decrease in circulating vitamin A levels to undetectable levels. WT and *Cd36*^*−/−*^ mice were placed on either a vitamin A sufficient (VAS) diet or vitamin A deficient (VAD) diet for 3-months immediately after weaning. Similar to our previous experiment, *Cd36*^*−/−*^ mice had reduced body mass, WAT mass, similar absolute liver mass, and relatively larger livers when normalized to body weight compared to WT mice (Table 2). These effects were not altered by consuming diets with varying vitamin A content indicating that any differences in physical characteristics were due to genotype and not dietary vitamin A content.

**Table 2:** Physical characteristics of WT and *Cd36*^*−/−*^ mice consuming diets with varying vitamin A content.

	Body weight (g)^a^	Liver weight (g)	Liver:body weight ratio^a^	White adipose weight (g)^a^	White adipose:body ratio^a,b^
**VAS**					
Wild type (7)	33.05 ± 4.76	1.41 ± 0.17	0.043 ± 0.001	1.81 ± 0.43	0.054 ± 0.006
*Cd36*^*−/−*^ (6)	29.71 ± 2.72	1.55 ± 0.19	0.052 ± 0.003	0.82 ± 0.18*	0.027 ± 0.004*
**VAD**					
Wild type (8)	36.18 ± 5.86	1.49 ± 0.38	0.041 ± 0.006	1.70 ± 0.39	0.047 ± 0.005
*Cd36*^*−/−*^ (5)	30.73 ± 3.49	1.55 ± 0.56	0.050 ± 0.14	0.78 ± 0.23*	0.025 ± 0.005*

The *n* number for each experimental group is provided in parentheses in the first column. All data are presented as mean ± SD. Data analyzed by two-way ANOVA.

*Post-test significantly different between genotypes within mice consuming the same diet.

^a^Significant genotype effect.

^b^Significant diet effect.

**Figure 3:**
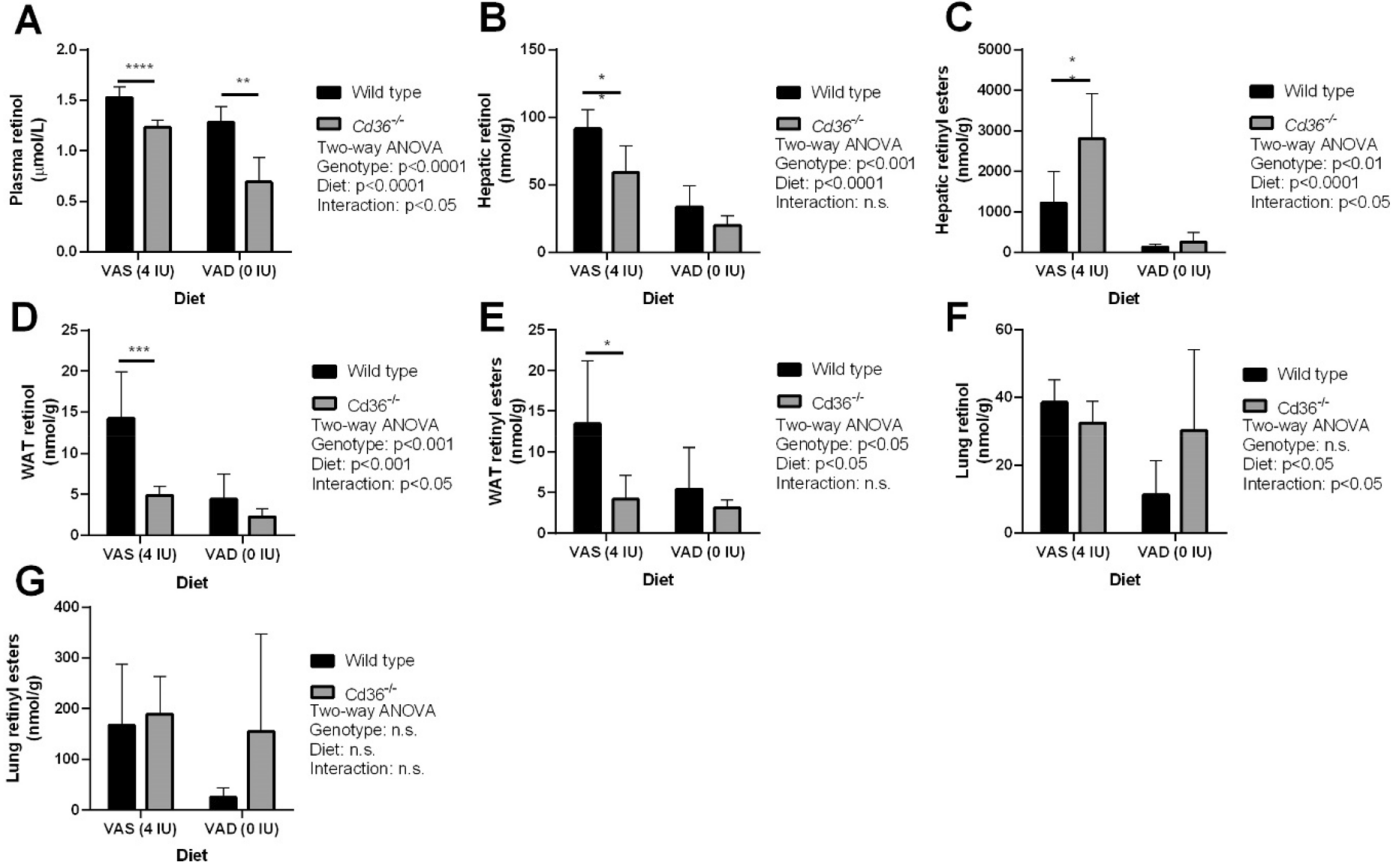
Tissue retinoid levels of wild type and *Cd36*^*−/−*^ mice on diets with varying vitamin A content. HPLC was used to determine tissue levels of (**A**) Plasma retinol, (**B**) Hepatic retinol, (**C**) Hepatic retinyl esters, (**D**) WAT retinol, (**E**) WAT retinyl esters, (**F**) Lung retinol, and (**G**) Lung retinyl esters in mice placed on a VAD diet from weaning. Tissue retinoid levels were normalized to volume of plasma (**A**) or tissue mass (**B**–**G**). Data is shown as mean ± S.D. and significance determined by two-way ANOVA (n = 6 per group). *p < 0.05, **p < 0.01, and ****p < 0.0001 denote a significant post-test result between genotypes within mice of the same age.

HPLC analysis of tissue retinoid levels revealed that *Cd36*^*−/−*^ mice were able to maintain circulating retinol levels that closely mimicked the drop in circulating retinol levels seen in WT mice placed on a VAD diet, although still slightly lower than WT mice (Fig. 3A). Additionally, *Cd36*^*−/−*^ mice displayed a similar reduction in hepatic retinoid levels on a VAD diet to WT mice (Fig. 3B,C). Analysis of WAT retinoid levels demonstrated that on a VAD diet there was a diet-dependant decrease in retinol levels and *Cd36*^*−/−*^ mice had significantly lower retinoid levels compared to WT mice (Fig. 3D,E). Similar to our aging study lung retinoid levels were similar between WT and *Cd36*^*−/−*^ mice, however the VAD diet induced a significant decrease in lung retinol levels (Fig. 3F) while retinyl ester levels were unaffected (Fig. 3G). In combination, these data suggest that *Cd36*^*−/−*^ mice are able to efficiently mobilize hepatic vitamin A arguing against a critical role of CD36 in hepatic vitamin A mobilization.

**Figure 4:**
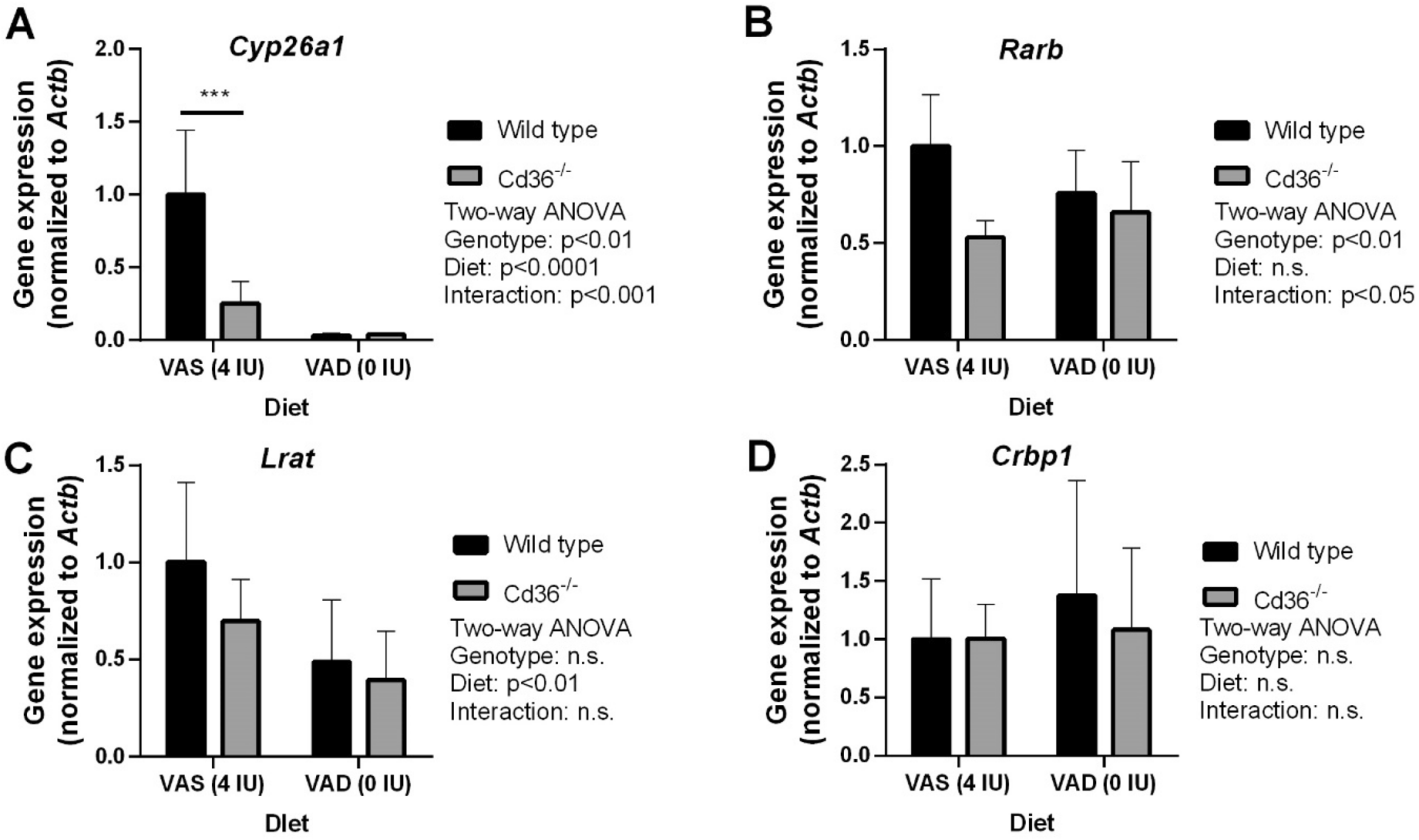
Hepatic gene expression levels of key mediators of vitamin A metabolism in wild type and *Cd36*^*−/−*^ mice on diets with varying vitamin A content. qPCR was used to determine relative hepatic gene expression levels of (**A**) *Cyp26a1*, (**B**) *Rarb*, (**C**) *Lrat*, (**D**) *Crbp1*. Data is shown as mean ± S.D. and significance determined by two-way ANOVA (n = 6 per group). *p < 0.05 denotes a significant post-test result between genotypes within mice of the same age.

To further investigate the impact of CD36 deficiency on hepatic retinoid metabolism on a VAD diet we determined gene expression levels of the same subset of genes explored earlier. Interestingly, on a VAS diet hepatic *Cyp26a1* expression was significantly lower in *Cd36*^*−/−*^ compared to WT mice (Fig. 4A). Irrespective of genotype, hepatic *Cyp26a1* followed hepatic vitamin A levels and was significantly decreased in both WT and *Cd36*^*−/−*^ mice on a VAD diet (Fig. 4A). Additionally, *Rarb* expression was lower in *Cd36*^*−/−*^ mice but was not affected by dietary vitamin A content (Fig. 4B). Hepatic *Lrat* expression was decreased in both WT and *Cd36*^*−/−*^ mice on a VAD diet (Fig. 4C) while *Crbp1* expression similar between WT and *Cd36*^*−/−*^ irrespective of dietary vitamin A content (Fig. 4D).

A possible limitation of our initial VAD feeding study was that the mice were placed on a VAD diet immediately following weaning. At this age, both WT and *Cd36*^*−/−*^ mice would not have established significant hepatic vitamin A stores, which would become more easily depleted when consuming a VAD diet, leading to lower plasma retinol levels in both strains as observed, and possibly masking an effect of *Cd36* deletion. By measuring plasma retinol levels in mice that were placed on a VAD diet at 3 months of age, we observed that plasma retinol levels significantly dropped in both WT and *Cd36*^*−/−*^ mice (see Supplementary Fig. S1 online), and consistent with our initial study *Cd36*^*−/−*^ mice maintained their ability to defend their circulating retinol levels. Additionally, liver, WAT, and lung tissue retinoid levels displayed a similar pattern to our initial dietary study (see Supplementary Fig. S1 online).

### *Cd36*^*−/−*^ mice have impaired circulating retinyl ester metabolism

To further explore the altered systemic vitamin A phenotype in *Cd36*^*−/−*^ mice we investigated the metabolism of circulating retinyl esters. Although the preponderance of circulating retinoid is in the form of retinol bound to RBP4, retinyl esters are the primary transport form of postprandial vitamin A in chylomicrons and are also secreted from the liver in very-low-density lipoprotein (VLDL) particles^9,10^.

**Figure 5:**
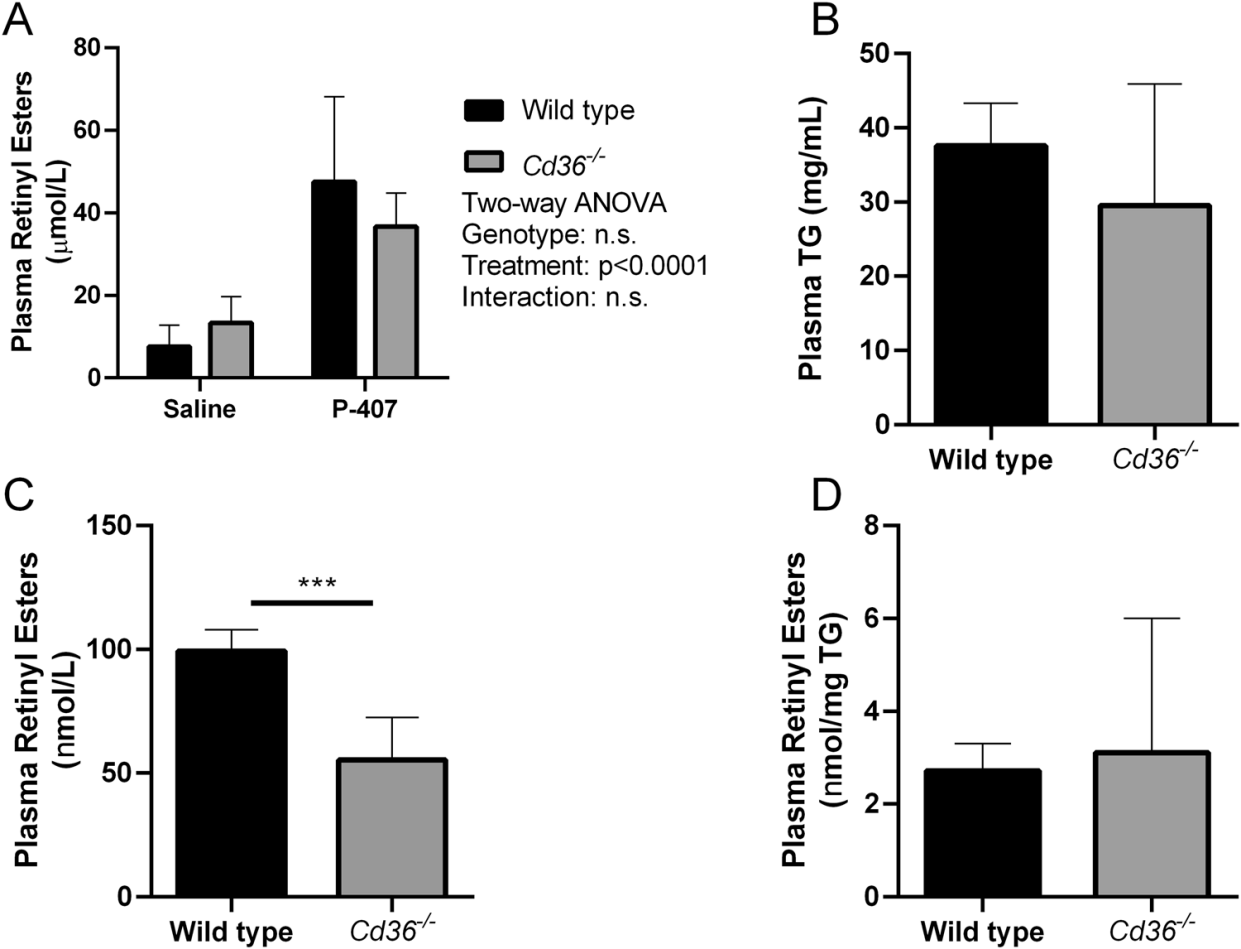
Circulating triglyceride and retinyl ester metabolism in wild type and *Cd36*^*−/−*^ mice. Postprandial (**A**) Postprandial plasma retinyl ester clearance was determined in saline and p-407 injected mice (WT Saline, n = 7; *Cd36*^*−/−*^ Saline, n = 6; WT p-407, n = 7; *Cd36*^*−/−*^ p-407, n = 5 mice per group). Hepatic VLDL **B**) triglyceride secretion and (**C**) retinyl ester secretion were determined in p-407 injected mice. (**D**) retinyl esters were normalized to triglyceride (n = 6 mice per group). Data is shown as mean ± S.D. and significance determined by two-way ANOVA (**A**) or Student’s t-test (**B**–**D**; ***p < 0.001).

To examine the clearance of postprandial retinyl esters, WT and *Cd36*^*−/−*^ mice were given an oral bolus of a pharmacologic dose of retinol. Additionally, both WT and *Cd36*^*−/−*^ mice were given an intraperitoneal injection of either saline or the lipase inhibitor poloxamer-407 (p-407). As expected, p-407 was associated with a significant increase in plasma retinyl ester levels but there was no effect of *Cd36* deficiency on postprandial retinyl ester clearance (Fig. 5A). These data suggest that absence of CD36 does not significantly impair intestinal incorporation and secretion of dietary retinol into chylomicrons.

Although generally regarded as a minor contributor to circulating retinoid levels, retinyl esters are released from the liver in VLDL particles^10^. *Cd36*^*−/−*^ mice have been previously reported to have reduced VLDL secretion^34^, thus reduced VLDL secretion may contribute to the accumulation of hepatic retinoid observed in *Cd36*^*−/−*^ mice. To examine this effect both WT and *Cd36*^*−/−*^ mice were subjected to an overnight fast and then treated with p-407 to cause accumulation of circulating VLDL particles. There was no difference in circulating TG levels between WT and *Cd36*^*−/−*^ mice (Fig. 5B) and reduced circulating retinyl esters when normalized to plasma volume (Fig. 5C). When circulating retinyl esters levels were normalized to TG there was no difference in circulating retinyl esters between WT and *Cd36*^*−/−*^ mice suggesting that incorporation of retinyl esters into VLDL particles is similar between the two genotypes (Fig. 5D).

## Discussion

In this study we set out to examine the role of CD36 in hepatic and whole-body vitamin A homeostasis. Although the role of CD36 has been well studied in several other physiological processes^15^, to our knowledge the role of CD36 has not been thoroughly investigated in vitamin A metabolism. Using observations from our previous work that *Cd36*^*−/−*^ mice retained hepatic vitamin A stores when fed alcohol^13,14^, we hypothesized that CD36 would have a direct role in hepatic vitamin A mobilization and loss of CD36 would render mice unable to mobilize hepatic vitamin A. We also postulated that an impaired ability of the liver to mobilize hepatic vitamin A would result in indirect changes to vitamin A levels in other tissues. The data from this study argue against CD36 being required for hepatic vitamin A mobilization; however, our data clearly demonstrate that absence of CD36 alters systemic vitamin A homeostasis, and suggest a role for CD36 in the postprandial clearance of chylomicron retinyl esters into WAT.

To investigate a direct role for CD36 in hepatic retinoid metabolism we examined hepatic vitamin A metabolism throughout the life span of WT and *Cd36*^*−/−*^ mice (Figs. 1 and 2). The results from these experiments supported our hypothesis as *Cd36*^*−/−*^ mice accumulated hepatic retinoid with age and had reduced circulating retinol levels compared to WT mice. Indeed, our hepatic gene expression data also supported this hypothesis, with aged *Cd36*^*−/−*^ mice having higher *Cyp26a1* expression suggesting some of the excess vitamin A was being catabolized to mitigate further hepatic accumulation of the excess retinoids within the liver. To further test this hypothesis, we manipulated hepatic vitamin A content by introducing conditions that would promote hepatic vitamin A mobilization (i.e. a VAD diet). We hypothesized that if CD36 was critical for mobilization of hepatic vitamin A then on a VAD diet *Cd36*^*−/−*^ mice would display a rapid drop in circulating retinol levels to undetectable levels while hepatic vitamin A remained unchanged. Conversely WT mice on a VAD diet would be able to maintain their circulating retinol levels mice while gradually drawing on (i.e. depleting) hepatic vitamin A stores^10^. Contrary to our hypothesis *Cd36*^*−/−*^ mice were able to maintain circulating retinol levels, albeit still slightly lower than WT mice, when placed on a VAD diet. Furthermore, hepatic vitamin A levels were similarly decreased in both WT and *Cd36*^*−/−*^ mice (Fig. 3). Our hepatic gene expression analysis in these mice revealed that *Cyp26a1* was significantly decreased in *Cd36*^*−/−*^ mice consuming a VAS diet, and that with depletion of hepatic vitamin A content the expression of this enzyme was reduced in both genotypes of mice. In our aging study, comparably aged *Cd36*^*−/−*^ mice (i.e. the 3-month time point) also tended to have decreased hepatic *Cyp26a1* expression. These data are consistent and highlight the contribution of CYP26A1 to homeostatic control of hepatic vitamin A levels^31^. With excess hepatic vitamin A accumulation (i.e. aged *Cd36*^*−/−*^ mice) this enzyme is upregulated to help dispose of vitamin A, whereas with depletion of hepatic vitamin A content (i.e. in mice consuming a VAD diet), this enzyme is downregulated.

Taken together, although our data indicate that CD36 is not required for hepatic vitamin A mobilization our data clearly illustrate that global CD36 deletion alters systemic vitamin A homeostasis. Notably, we observed a significant decrease in WAT retinoid levels in *Cd36*^*−/−*^ mice compared to WT mice (Figs. 1 and 3). We think this observation is of central importance to understand hepatic retinoid accumulation in *Cd36*^*−/−*^ mice. Upon further investigation of previous reports using *Cd36*^*−/−*^ mice it is evident that chylomicron metabolism is altered compared to WT mice. Surprisingly, despite the well characterized role of CD36 in FA uptake *Cd36*^*−/−*^ mice do not have impaired intestinal fat absorption, per se, but instead absorption shifts from the proximal to distal portion of the intestine^16,35^. Additionally, CD36 deficiency results in a delayed postprandial chylomicron secretion from the intestine, and reduced circulating chylomicron clearance^36^. The delayed chylomicron clearance has been attributed to the inhibitory action of increased circulating FA levels impairing the action of lipoprotein lipase (LPL). Although not as dramatic as previous reports our data further support that *Cd36*^*−/−*^ mice have delayed chylomicron TG clearance (Fig. 5). This led to a new working hypothesis that the altered vitamin A phenotype in *Cd36*^*−/−*^ mice is due to the indirect effect of impaired chylomicron metabolism. Combining our data of decreased WAT retinoid levels, increased hepatic retinoid levels, and previous reports of impaired chylomicron metabolism, it is possible to speculate that postprandial chylomicron retinyl esters that would normally be taken up by WAT in WT mice are redirected to the liver in chylomicron remnants in *Cd36*^*−/−*^ mice. There is precedence for this, van Bennekum et al. previously showed that heparinized rats exhibit increased postprandial hepatic retinyl ester uptake^37^. Although in the opposite direction, it has also been shown that in *Lrat*^*−/−*^ mice the inability to store retinoids in the liver leads to increased flux and storage in WAT^6^. Thus, it seems that the liver and WAT can undergo compensatory increases in their ability to store vitamin A when the other tissue’s ability to store vitamin A is impaired. To understand the underlying mechanism of impaired uptake of postprandial chylomicron retinyl ester in the WAT of *Cd36*^*−/−*^ mice, it is important to consider the well-described coupling of LPL and CD36 in the clearance of chylomicron TGs^19^. The current paradigm proposes that LPL hydrolyses chylomicron TG and CD36 facilitates the uptake of liberated FAs. Interestingly, LPL has previously been shown to hydrolyze retinyl esters in addition to TG^38^. Thus, one possible interpretation is that impaired LPL activity in *Cd36*^*−/−*^ mice results in reduced retinyl ester hydrolysis and subsequent uptake by WAT. Alternatively, CD36 itself may facilitate retinol uptake into adipose tissue, and in its absence this pathway is blunted. While we prefer the former putative mechanism, we have no direct evidence to support either interpretation. We were unable to detect a significant difference in circulating postprandial retinyl ester levels between WT and *Cd36*^*−/−*^ mice, however it is possible that our assay was not sensitive enough or was statistically underpowered to detect differences between genotypes. Furthermore, we did not directly measure tissue uptake of postprandial retinyl esters in either WAT or liver, thus this proposed mechanism will require further investigation.

Although a relatively minor contributor to hepatic vitamin A secretion in humans and rodents, retinyl esters are incorporated into VLDL particles and secreted by the liver^10^. It has previously been reported that *Cd36*^*−/−*^ mice have reduced VLDL secretion compared to WT animals^34^. Interestingly, while we did not observe decreased fasting TG levels in *Cd36*^*−/−*^ mice, retinyl ester levels were decreased (Fig. 5). Nevertheless, when circulating fasting retinyl ester levels were normalized to circulating TG, there was no difference between WT and *Cd36*^*−/−*^ mice. While this data suggests that decreased hepatic retinyl ester secretion would further contribute to hepatic retinoid accumulation in *Cd36*^*−/−*^ mice, further investigation of VLDL-retinyl ester secretion in *Cd36*^*−/−*^ mice is required.

One question our data, and our new working hypothesis, has not specifically addressed is the tendency for *Cd36*^*−/−*^ mice to accumulate hepatic retinyl esters while having lower hepatic retinol compared to WT mice on a diet with adequate vitamin A content. The current paradigm of hepatic retinoid flux is that when acquired by hepatocytes chylomicron remnant retinyl ester is hydrolyzed to retinol, transferred to HSCs, converted to retinyl esters, and stored within HSCs localized to specialized lipid droplets^7,10^. When hepatic vitamin A stores are required to satisfy peripheral demand HSC retinyl esters are hydrolyzed to retinol, transferred to hepatocytes, and then secreted into circulation bound to RBP4^8–10^. The increased proportion of retinyl esters compared to retinol would indicate that the flux of postprandial retinoid from hepatocytes to HSCs is unimpaired. Indeed, hepatocytes are unable to store significant quantities of retinyl esters^39^. The accumulation of retinyl esters would suggest that in *Cd36*^*−/−*^ mice the excess retinoid is being trapped in HSCs. This may be due to an impaired ability to transfer retinol from HSCs to hepatocytes or an impaired ability to hydrolyze retinyl esters within HSCs. Despite decades of thorough investigation, the proteins mediating the transfer of retinol between hepatocytes and HSCs (and vice-versa) are unknown^10^. Additionally, the identity of the enzyme, or enzymes, responsible for retinyl ester hydrolysis are unknown. Several lipases have shown the ability to hydrolyze retinyl esters however a sole enzyme responsible for retinyl hydrolysis has not been established^40–43^. Recently, a report implicating pancreatic lipase related protein 2 (PLRP2) was proposed to be the primary retinyl ester hydrolase; however, when *Plrp2*^*−/−*^ mice were placed on the VAD diet they were still able to mobilize hepatic retinoid, thus PLRP2 is not the sole retinyl ester hydrolase. To our knowledge hepatic lipolysis has not been investigated in *Cd36*^*−/−*^ mice however in adipocytes lipolysis is increased compared to WT mice^44^. The tendency for *Cd36*^*−/−*^ mice to accumulate more retinyl esters in the liver while having reduced retinol levels compared to WT will require further investigation.

In conclusion, we demonstrate that the absence of CD36 alters whole-body vitamin A homeostasis. We also provide evidence that CD36 is not required for hepatic retinoid mobilization, however our data do not eliminate the possibility that CD36 has a direct role in hepatic retinoid metabolism. While the precise mechanism for this altered phenotype is still under investigation, we propose that the altered systemic retinoid phenotype in *Cd36*^*−/−*^ mice is due to impaired chylomicron metabolism that manifests as a redirection of postprandial vitamin A from WAT to the liver.

## Materials and methods

### Animals

All animal experiments were conducted in accordance with the guidelines established by The Canadian Council on Animal Care and approved by the University of Alberta Animal Research Ethics Committee. All experiments were conducted using WT and *Cd36*^*−/−*^ mice in a congenic C57BL/6 background and were generated as previously described^16^. Specialized research diets were obtained from Bio-Serv (Flemington, NJ, USA). The VAS diet contained 4 IU/g vitamin A in the form of retinyl palmitate and the VAD diet contained 0 IU/g vitamin A. In aging experiments weaned animals were collected directly from their home cage 20 days after birth, and dams were allowed access to a standard rodent breeder chow diet ad libitum (14 IU/g vitamin A).

### Tissue collection

Tissues were collected in the morning, without fasting, between 09:00–10:00. Animals were anesthetized by isoflurane and then euthanized by cervical dislocation. Blood was obtained via cardiac puncture, plasma was separated by centrifugation, placed in a clean tube, and then snap frozen in liquid N_2_. Epididymal WAT, lungs, and liver were dissected, weighed, placed in a clean tube, and snap frozen in liquid N_2_. Tissues were stored at -80 °C until used in analysis.

### Analysis of tissue retinoid content

HPLC was used to measure tissue vitamin A levels (i.e. retinol and retinyl ester) in all tissues examined using standard methods^45^. Briefly, retinoids were extracted from tissue homogenates in hexanes with retinyl acetate (Sigma-Aldrich, Oakville, ON, CA) as an internal standard. Extracted samples were evaporated under a gentle stream of nitrogen and dissolved in mobile phase (70% v/v acetonitrile, 15% v/v methanol, 15% v/v methylene chloride). Samples were analyzed using an Agilent 1200 HPLC system (Santa Clara, CA, United States) running ChemStation software (Agilent), with a Zorbax Eclipse Plus C18 separating column (4.6 × 250 mm, 5 μm particle size; Agilent). Extracted retinoid concentrations were calculated by measuring the area under the curve of absorbance peaks at 325 nm and adjusted based on the recovery of the internal standard.

### Gene expression analysis

Tissue gene expression was determined using quantitative PCR (qPCR). RNA was extracted from tissues using TRIzol (Invitrogen, Burlington, ON, CA), according to the manufacturer’s protocol. RNA clean-up was performed on a Qiagen (Toronto, ON, CA) RNeasy plus column. Concentration and quality of extracted RNA were determined using a NanoDrop1000 spectrophotometer (Thermo-Fisher Scientific, Mississauga, ON, CA). Two micrograms of purified RNA were reverse-transcribed into cDNA using a high-capacity cDNA Reverse Transcription kit (Applied Biosystems, Burlington, ON, CA). Quantitative PCR was performed using a QuantStudio3 (Applied Biosystems) with SYBR Green PCR master mix (Roche Diagnostics, Mississauga, ON, CA) under uniform reaction conditions containing 5 μl of cDNA and 15 μl of master mix, containing gene specific primers (Table 3). Expression of target genes was normalized to *Actb* and relative gene expression levels were determined based on the cycle threshold of amplified genes and calculated using a modified method described by Pfaffl^46^.

**Table 3:** List of genes and primers sequences used in qPCR.

Gene name	Primer sequence	Amplicon size
*Actb*	Forward: AGCTATGAGCTGCCTGACG Reverse: TGCCACAGGATTCCATACCCAAG	106
*Cyp26a1*	Forward: GGCACTGTGATTGGCAGCTTCTAA Reverse: TGCAGGGAGATTGTCCACAGGGTA	73
*Rarb*	Forward: GGGCATGTCCAAAGAGTCTGTTAG Reverse: CTAGCTCCGCTGTCATCTCATAG	101
*Lrat*	Forward: CAGGCATCGAAGAGATGACTCCG Reverse: GCTGCTGGTAACTAAATCCTGGTCC	84
*Crbp1*	Forward: CTTACTGTCCCTACTGTGTGTCAAGCACTA Reverse: CCTGAGATGAACCTCCTGAGATGGTTTA	73

### Postprandial retinyl ester clearance

Mice were fasted for 12 h starting at 21:00 and then received an oral bolus containing 1 mg of all-trans retinol (Sigma-Aldrich, R7632) in 250 μl of walnut oil at 09:00 the following morning. Mice were then sacrificed 4 h post-gavage and plasma was collected as described above. Where indicated, mice received an intraperitoneal injection of the lipase inhibitor p-407 (1 g/kg body weight; Sigma-Aldrich). Plasma retinyl ester levels were determined as described above.

### Analysis of VLDL secretion

Mice were fasted for 12 h starting at 21:00. A blood sample was collected from the tail vein at 09:00 the following morning and then mice received an intraperitoneal injection of p-407 (1 g/kg body weight). Mice were sacrificed 4 h post-gavage and plasma was collected via cardiac puncture as described above. Plasma TG levels were determined using an Infinity TG liquid stable reagent according to the manufacturer’s protocol (Thermo-Fisher Scientific), using an Epoch2 micro plate reader (Bio-Tek, San Diego, CA, USA). Plasma retinoid levels were determined as above.

### Statistical analysis

All data were analyzed using Prism 8 (GraphPad, San Diego, CA, USA), and presented as the mean ± standard deviation (SD). A two-way ANOVA with a Bonferroni post-test or a Student’s t-test was used to determine statistical differences between groups. In all analyses, a p-value < 0.05 was considered statistically significant.

## Supplementary information

Supplementary information.

## Data Availability

The datasets generated and analyzed during this work are available upon written request to the corresponding author (R.D.C.).
