# Author Correction: Absence of CD36 alters systemic vitamin A homeostasis

**DOI:** 10.1038/s41598-025-02637-0

**Published:** 2025-06-16

**Authors:** Michael J. Trites, Maria Febbraio, Robin D. Clugston

**Affiliations:** 1 Department of Physiology, University of Alberta, 7‑49 Medical Sciences Building, Edmonton, AB T6G 2H7 Canada; 2 Group on the Molecular and Cell Biology of Lipids, Faculty of Medicine and Dentistry, University of Alberta, Edmonton, AB Canada; 3 Department of Dentistry, Faculty of Medicine and Dentistry, University of Alberta, Edmonton, AB Canada

Correction to: *Scientific Reports* 10.1038/s41598-020-77411-5, published online 23 November 2020

The original version of this Article contained errors.

The plasma triglyceride values reported in Fig. 5A and 5D were miscalculated. The plasma retinyl ester values normalized to these plasma triglyceride values, in Fig. 5C and Fig. 5F respectively, were therefore also incorrect. In reanalysing the data, it was found that the sample size for Figure 5A and Figure 5C was insufficient, and these plots have been removed. As a result, the figure legend has been corrected. The original version of Fig. 5 and accompanying original legend appear below.

**Fig. 5:**
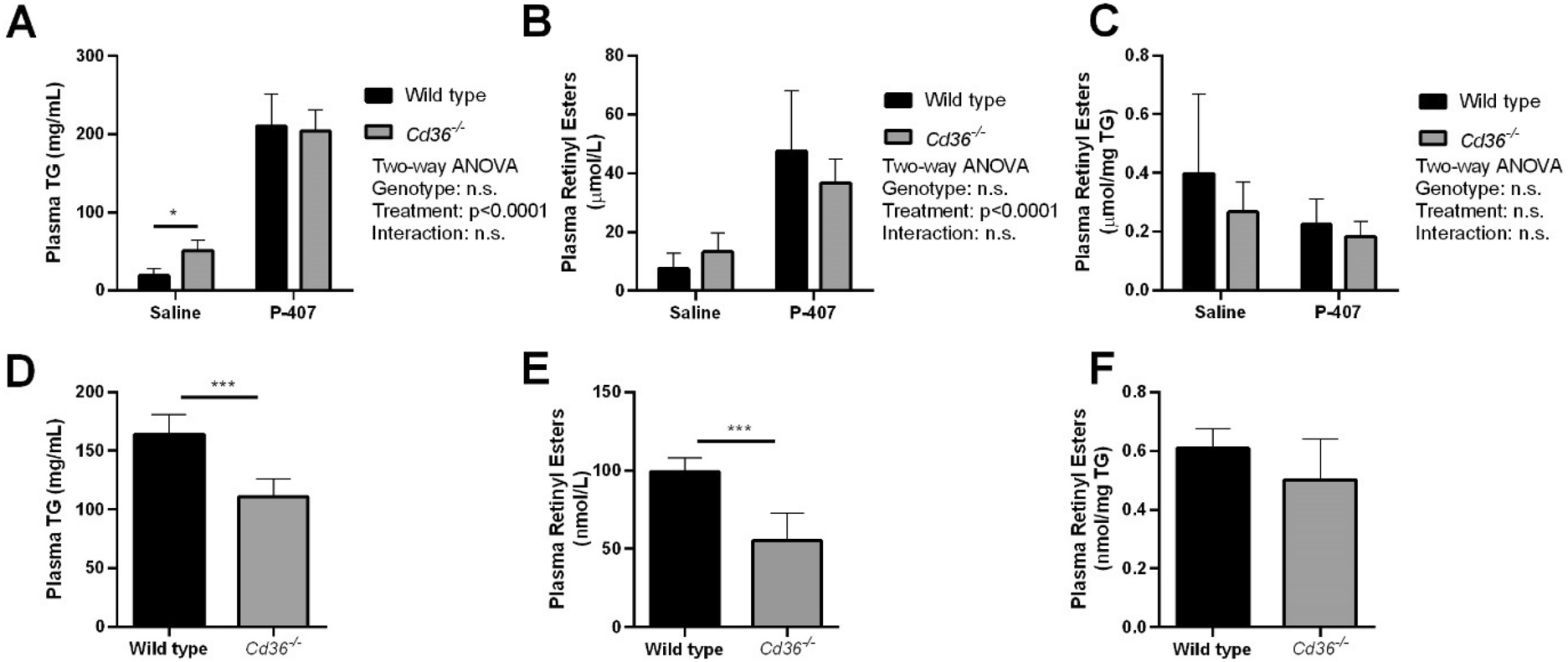
Circulating triglyceride and retinyl ester metabolism in wild type and *Cd36*^*-/-*^ mice. Postprandial A) plasma triglyceride and B) plasma retinyl ester clearance was determined in saline and p-407 injected mice. C) Retinyl esters were also normalized to plasma TG (WT Saline, n = 7; *Cd36*^*-/-*^ Saline, n = 6; WT p-407, n = 7; *Cd36*^*-/-*^ p-407, n = 5 mice per group). Hepatic VLDL D) triglyceride secretion and E) retinyl ester secretion were determined in p-407 injected mice. F) retinyl esters were normalized to triglyceride (n = 6 mice per group). Data is shown as mean ± S.D. and significance determined by two-way ANOVA (A-C; *p < 0.05 denotes a significant post-test result between genotypes within the same treatment) or Student’s t-test (D-F; ***p < 0.001).

Given these changes, the text in the Abstract,

“In conclusion, we demonstrate that the absence of CD36 alters whole-body vitamin A homeostasis and suggest that this phenotype is secondary to the impaired chylomicron metabolism previously reported in these mice.”

now reads:

“In conclusion, we demonstrate that the absence of CD36 alters whole-body vitamin A homeostasis.”

In addition, in the Results section, under the subheading ‘*Cd36*^*-/-*^ mice have impaired circulating retinyl ester metabolism’,

“Consistent with previous reports, *Cd36*^*−/−*^ mice had delayed triglyceride (TG) clearance when treated with saline but not with p-407, suggesting that *Cd36*^*−/−*^ mice have delayed postprandial chylomicron clearance and impaired peripheral lipase activity (Fig. 5A)^34,35^. However, this did not manifest in reduced postprandial retinyl ester clearance when normalized to plasma volume (Fig. 5B) or reduced retinyl ester clearance when normalized to TG (Fig. 5C). These data also suggest that absence of CD36 does not significantly impair intestinal incorporation and secretion of dietary retinol into chylomicrons.”

now reads:

“As expected, p-407 was associated with a significant increase in plasma retinyl ester levels but there was no effect of *Cd36* deficiency on postprandial retinyl ester clearance (Fig. 5A). These data suggest that absence of CD36 does not significantly impair intestinal incorporation and secretion of dietary retinol into chylomicrons.”

And,

“*Cd36*^*−/−*^ mice had reduced circulating TG compared to WT mice (Fig. 5D) and reduced circulating retinyl esters when normalized to plasma volume (Fig. 5E). When circulating retinyl esters levels were normalized to TG there was no difference in circulating retinyl esters between WT and *Cd36*^*−/−*^ mice suggesting that incorporation of retinyl esters into VLDL particles is similar between the two genotypes (Fig. 5F).

now reads:

“There was no difference in circulating TG levels between WT and *Cd36*^*−/−*^ mice (Fig. 5B) and reduced circulating retinyl esters when normalized to plasma volume (Fig. 5C). When circulating retinyl esters levels were normalized to TG there was no difference in circulating retinyl esters between WT and *Cd36*^*−/−*^ mice suggesting that incorporation of retinyl esters into VLDL particles is similar between the two genotypes (Fig. 5D).”

In the Discussion,

“Combining our data of decreased WAT retinoid levels, increased hepatic retinoid levels, delayed postprandial TG clearance, and previous reports of impaired chylomicron metabolism, it is possible that postprandial chylomicron retinyl esters that would normally be taken up by WAT in WT mice are redirected to the liver in chylomicron remnants in *Cd36*^*−/−*^ mice.”

now reads:

“Combining our data of decreased WAT retinoid levels, increased hepatic retinoid levels, and previous reports of impaired chylomicron metabolism, it is possible to speculate that postprandial chylomicron retinyl esters that would normally be taken up by WAT in WT mice are redirected to the liver in chylomicron remnants in *Cd36*^*−/−*^ mice.”

And,

“Consistent with previous reports we observed that *Cd36*^*−/−*^ mice had reduced VLDL secretion compared to WT animals and this correlated with decreased fasting retinyl ester levels (Fig. 5)^36^. When circulating fasting retinyl ester levels were normalized to circulating TG there was no difference between WT and *Cd36*^*−/−*^ mice suggesting that retinyl ester incorporation into VLDL particles is similar between these two genotypes. Thus, decreased VLDL-retinyl ester secretion could further contribute to hepatic retinoid accumulation in *Cd36*^*−/−*^ mice.”

now reads:

“It has previously been reported that Cd36-/- mice have reduced VLDL secretion compared to WT animals^34^. Interestingly, while we did not observe decreased fasting TG levels in *Cd36*^*-/-*^ mice, retinyl ester levels were decreased (Fig. 5). Nevertheless, when circulating fasting retinyl ester levels were normalized to circulating TG, there was no difference between WT and *Cd36*^*-/-*^ mice. While this data suggests that decreased hepatic retinyl ester secretion would further contribute to hepatic retinoid accumulation in *Cd36*^*-/-*^ mice, further investigation of VLDL-retinyl ester secretion in *Cd36*^*-/-*^ mice is required.”

In the Methods section, the subheading “Postprandial plasma triglyceride and retinyl ester clearance” has been changed to “Postprandial retinyl ester clearance”, and

*“*Mice were then sacrificed 4 hours post-gavage and plasma was collected as described above. Where indicated, mice received an intraperitoneal injection of the lipase inhibitor p-407 (1 g/kg body weight; Sigma-Aldrich). TG levels were determined using an Infinity TG liquid stable reagent according to the manufacturer’s protocol (Thermo-Fisher Scientific), using an Epoch2 micro plate reader (Bio-Tek, San Diego, CA, USA). Plasma retinyl ester levels were determined as described above.”

now reads:

“Mice were then sacrificed 4 hours post-gavage and plasma was collected as described above. Where indicated, mice received an intraperitoneal injection of the lipase inhibitor p-407 (1 g/kg body weight; Sigma-Aldrich). Plasma retinyl ester levels were determined as described above.”

In the Methods section, under the subheading “Analysis of VLDL secretion”,

“Plasma TG and retinoid levels were determined as described above.”

now reads:

“Plasma TG levels were determined using an Infinity TG liquid stable reagent according to the manufacturer’s protocol (Thermo-Fisher Scientific), using an Epoch2 micro plate reader (Bio-Tek, San Diego, CA, USA). Plasma retinoid levels were determined as above.”

As a result of the above changes, former Reference 34, Drover, V. A. et al*.* CD36 deficiency impairs intestinal lipid secretion and clearance of chylomicrons from the blood. *J. Clin. Invest.* **115**, 1290–1297. https://doi.org/10.1172/jci21514 (2005), and Reference 35, Goudriaan, J. R. et al*.* CD36 deficiency in mice impairs lipoprotein lipase-mediated triglyceride clearance. *J. Lipid Res.* **46**, 2175–2181. https://doi.org/10.1194/jlr.M500112-JLR200 (2005), have been removed, and the subsequent references have been renumbered.

The original Article has been corrected.

Michael J. Trites disagrees with this correction.

