# Supplementary material for: Absence of CD36 alters systemic vitamin A homeostasis
Source: Sci Rep. 2020 Nov 23;10:20386. doi: 10.1038/s41598-020-77411-5 (PMC7683526; doi:10.1038/s41598-020-77411-5)
Supplement: Supplementary file 1 — Supplementary information. [file 41598_2020_77411_MOESM1_ESM.pdf]

Supplementary Information

Absence of CD36 alters systemic vitamin A homeostasis

Michael J. Trites, Maria Febbraio, and *Robin D. Clugston

*Corresponding author, Robin D. Clugston, E-mail: clugston@ualberta.ca

Supplementary Figure S1 Tissue retinoid levels of wild type and *Cd36$^{-/-}$* mice on diets with varying vitamin A content.

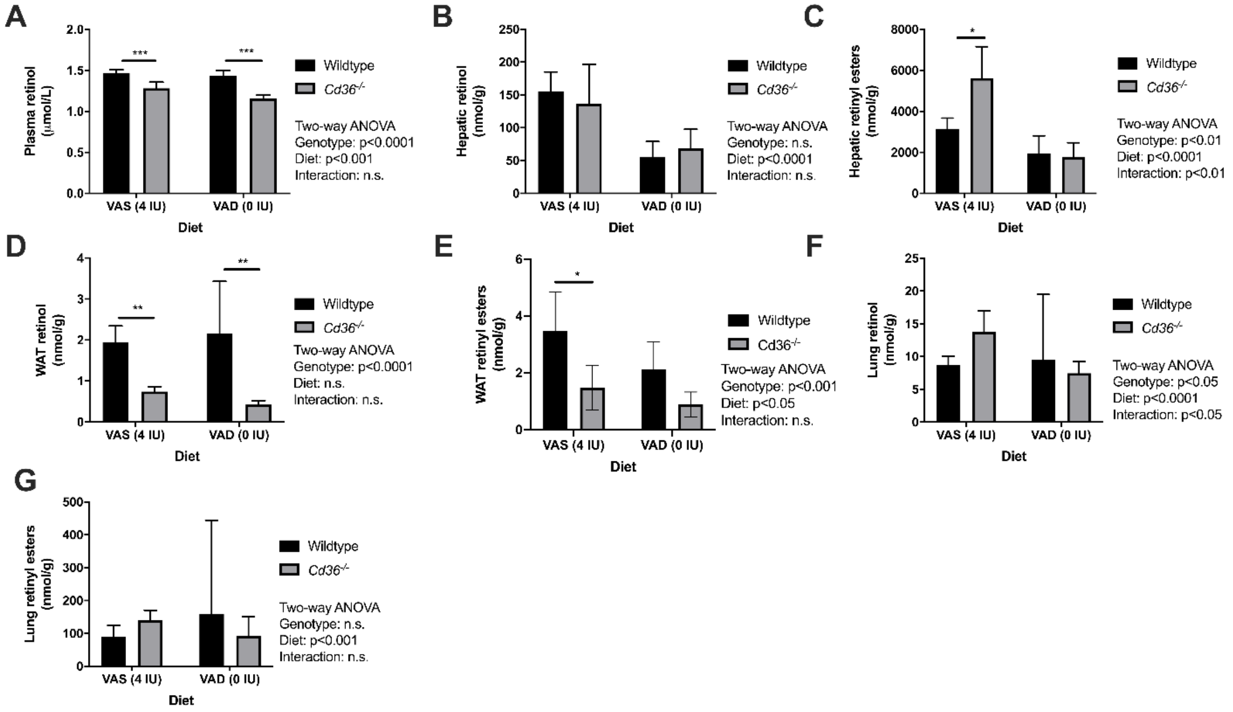

HPLC was used to determine tissue vitamin A levels in A) Plasma retinol, B) Hepatic retinol, C) Hepatic retinyl esters, D) WAT retinol, E) WAT retinyl esters, F) Lung retinol, and G) Lung retinyl esters in mice placed on a VAD diet from 3-months of age. Tissue retinoid levels were normalized to volume of plasma (A) or tissue mass (B-G). Data is shown as mean ± S.D. and significance determined by two-way ANOVA (n = 6 per group). *p<0.05, **p<0.01, and ***p<0.001 denote a significant post-test result between genotypes within mice of the same age.
